# Accelerometer-Based Fall Detection Using Machine Learning: Training and Testing on Real-World Falls

**DOI:** 10.3390/s20226479

**Published:** 2020-11-13

**Authors:** Luca Palmerini, Jochen Klenk, Clemens Becker, Lorenzo Chiari

**Affiliations:** 1 Department of Electrical, Electronic, and Information Engineering “Guglielmo Marconi”, University of Bologna, 40136 Bologna, Italy; luca.palmerini@unibo.it; 2 Health Sciences and Technologies—Interdepartmental Center for Industrial Research (CIRI-SDV), University of Bologna, 40126 Bologna, Italy; 3 Department of Clinical Gerontology, Robert-Bosch-Hospital, 70376 Stuttgart, Germany; Jochen.Klenk@rbk.de (J.K.); Clemens.Becker@rbk.de (C.B.); 4 Institute of Epidemiology and Medical Biometry, Ulm University, 89081 Ulm, Germany; 5 Study Centre Stuttgart, IB University of Applied Health and Social Sciences, 70178 Stuttgart, Germany

**Keywords:** accelerometer, fall detection, machine learning, wearable, smartphone

## Abstract

Falling is a significant health problem. Fall detection, to alert for medical attention, has been gaining increasing attention. Still, most of the existing studies use falls simulated in a laboratory environment to test the obtained performance. We analyzed the acceleration signals recorded by an inertial sensor on the lower back during 143 real-world falls (the most extensive collection to date) from the FARSEEING repository. Such data were obtained from continuous real-world monitoring of subjects with a moderate-to-high risk of falling. We designed and tested fall detection algorithms using features inspired by a multiphase fall model and a machine learning approach. The obtained results suggest that algorithms can learn effectively from features extracted from a multiphase fall model, consistently overperforming more conventional features. The most promising method (support vector machines and features from the multiphase fall model) obtained a sensitivity higher than 80%, a false alarm rate per hour of 0.56, and an F-measure of 64.6%. The reported results and methodologies represent an advancement of knowledge on real-world fall detection and suggest useful metrics for characterizing fall detection systems for real-world use.

## 1. Introduction

The topic of fall detection is gaining increasing attention and scientific contributions in the research community [1,2,3,4,5,6,7,8]. Falling is a significant health problem. Approximately 30% of people over 65 years of age living in the community fall each year [9]. Between 5% and 10% of all falls result in a fracture, and up to 90% of all fractures are caused by a fall [10]. Falls are also the cause of other adverse events, such as long lies with the inability to recover [11,12]. Therefore, effective automatic fall detection, to alert for medical attention, is of paramount importance.

A significant amount of research using wearable technology for automatic fall detection has been carried out in recent years. The subject usually wears wearable sensors in a fixed (e.g., waist belt) or semi-fixed (e.g., pendant) position. The recorded signals are usually kinematic signals (accelerations and angular velocities) and barometric pressure signals (which can estimate height change).

It is well known [1,2,4,13,14,15,16,17] that one of the main gaps in this research field is the practical impossibility to analyze real-world falls from older people for the validation (and, possibly, for the development) of fall detection algorithms. For several reasons, data relating to falls are challenging to obtain in a real-world setting, while data relating to activities of daily living (ADLs) are more straightforward to collect. Only a few works until now have used real-world falls for fall detection. The number of falls considered in these studies was limited, and most of these studies analyzed less than 30 falls [18,19,20,21,22,23,24,25].

Most fall detection studies used simulated falls (usually performed by young subjects) to create and validate the proposed fall detection systems. Such an approach leads to generalizability and transferability issues of the results from simulated to real environments, a topic covered in recent papers [24,26]. A simulated environment can indeed mask a series of possible problems that can disclose in real-world settings. Simulations are, in fact, performed in a controlled environment, which provides a standardized and clean version of the problem of fall detection. In contrast, in an uncontrolled environment, several procedural and technical issues need to be faced.

Chaudhuri et al. [15] demonstrated this issue’s prevalence by testing a wearable device for fall detection on 18 people. Fall detection was inaccurate, especially considering sensitivity (percentage of falls correctly detected). While the sensitivity reported by the manufacturer was between 94.1% and 94.4% (assessed using simulated falls from 59 people), the sensitivity evaluated on real-world falls dropped to a value of 25%. The authors conclude that “clinicians working with older adults need to assess for the availability (and accuracy) of real-world testing of any fall detection devices before recommending them to patients”.

Lipsitz et al. [16] recently tested a fall detection device based on a pendant sensor worn around the necks of 62 residents living in a nursing home. They evaluated its performance against the daily reports of healthcare staff. The device showed very poor sensitivity (only 19% of falls were correctly detected).

In a previous work [18], we proposed and validated a fall detection algorithm on real-world falls collected during the FARSEEING project [27]. However, the number of falls considered in that work was limited (29 falls). Since then, the number of falls in the FARSEEING fall repository [28] has grown. In the current work, we significantly expanded the number of analyzed real-world falls from 29 to 143. The increased number of falls allowed us to use machine-learning models to learn the characteristic patterns of falls better.

Several researchers have already used machine learning for fall detection [29]. Different classifiers have been used to detect falls, such as support vector machines (SVM) [19,25,30,31,32,33,34,35,36], Naïve Bayes [33,36,37], logistic regression [30,34,36], k-nearest neighbors (KNN) [31,33,34,36,38], decision trees and random forests [33,36,39], artificial neural networks, and deep learning [40,41,42]. However, in most of these studies, learning and testing were performed with simulated falls. This is the first work where machine learning algorithms learn from more than 100 real-world falls, to the best of our knowledge. 

Our objective in this paper is to analyze the performance of state-of-the-art machine learning algorithms on newly proposed features when benchmarked on a relatively large database of real-world falls. 

## 2. Methods

We analyzed the FARSEEING fall repository [28]. Falls in the repository were obtained from continuous monitoring of subjects using wearable sensors. Subjects were sampled from different populations with a moderate-to-high risk of falling (e.g., cerebellar and sensory ataxia, progressive supranuclear palsy, Parkinson’s disease) and monitored in different settings (e.g., community-dwelling, geriatric rehabilitation).

We used the FRAT-up tool [43] to compute these subjects’ average fall risk (the probability of experiencing a fall in the following year).

For each reported fall, the instant where the impact took place (impact sample, IS) was identified and annotated by two experts in signal interpretation, based on the reported time of fall, the description of the fall, and the characteristics of the recorded signal. A detailed description of the fall identification procedure has been published elsewhere [28]. We also analyzed activities of daily living (ADL), which correspond to recordings with no reported falls.

### 2.1. Recording Systems

An inertial sensor, either embedded on (i) smartphones or on (ii) dedicated systems, recorded the available signals. Both types of systems were body-fixed and worn on the lower back. The first type was secured in place by a waist-worn belt; the second type was attached to the skin with medical tape. In compliance with the FARSEEING consensus [44] on the recording of fall data, the inertial sensor always included at least a triaxial accelerometer with a full scale of at least ±2 g. All signals analyzed in this study had a sampling frequency of 100 Hz. Further details on the FARSEEING fall repository are available in [28].

### 2.2. Falls

We analyzed 143 fall recordings from 40 subjects. Most of the fall recordings were 40 min long: 20 min before and 20 min after IS. The sample population was 69.2 ± 12.7 years old (mean and standard deviation), and 55% (22/40) female.

### 2.3. Activities of Daily Living

We recorded the activities of daily living (ADLs) from 15 subjects, to compare and differentiate ADLs to/from falls. The ADL recordings were 12 h long and collected during daytime hours with the same types of systems described above. Hence, we analyzed a total of 180 h of ADL recordings (15 subjects × 12 h per subject).

The subjects with ADL recordings were 68.1 ± 15 years old, and 53% (8/15) female. Nine of these subjects also had falls analyzed in this study.

Reported falls were not present in any of the 15 ADL recordings.

### 2.4. Signal Processing

In this study, we focused on acceleration signals. All ADLs were recorded with sensors with a ±2 g range. All falls were recorded with the same range, except for sixteen falls (11.2%), which were recorded using a ±6 g range. Therefore, in order to process comparable signals, accelerations of falls recorded with the ±6 g range were normalized into the ±2 g range (i.e., if one of the acceleration signals was over 2 g (below −2 g) in one or more of its samples, it was set to 2 g (−2 g)).

We then computed the acceleration norm (also referred to as magnitude or sum vector in the scientific literature), which was obtained by the acceleration signals along the three sensing axes:

(1) $${Norm}_{acc}\left(t\right)=\sqrt{a_{x}{\left(t\right)}^{2}+a_{y}{\left(t\right)}^{2}+a_{z}{\left(t\right)}^{2}},$$

**Figure 1:**
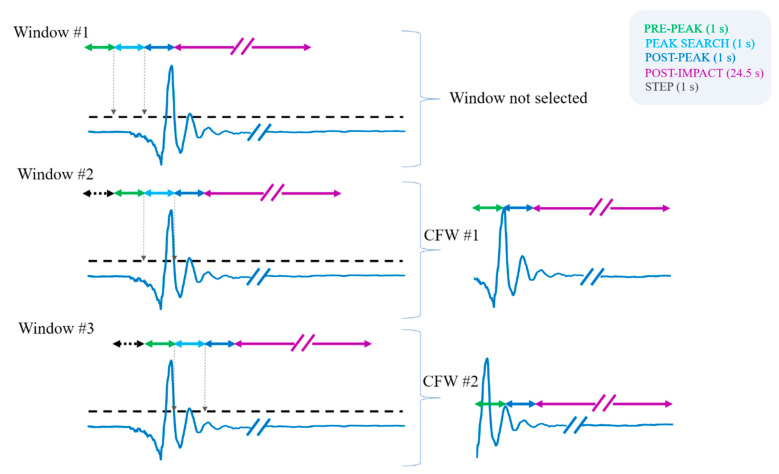
The windowing procedure is applied to a representative acceleration norm signal. The dashed line represents the 1.4 g threshold. Three consecutive windows are shown and analyzed. The first window is not selected, since no peaks are detected in the peak “search” interval. Windows #2 and #3 are instead two possible candidate fall windows (CFWs), and are then passed to the next steps for feature extraction and classification.

An ad hoc windowing technique (see Figure 1) analyzed the Norm_acc_ signal. We used overlapping windows to simulate the online functioning of a fall detection algorithm (even if the signal evaluation was performed offline) and to obtain a window for feature extraction with a precise time reference (with respect to the Norm_acc_ peak).

We used windows of 27.5 s, with a step of one second (or, equivalently, with a 96.4% overlap). The designed procedure provides a set of candidate fall windows (CFW) for further analysis that are all 26.5 s long. This duration reflects a multiphase fall model (slightly modified from [45]), with a pre-peak, a post-peak, and a post-impact phase (see examples of CFWs in the right side of Figure 1). Phases are defined based on the distance from the Norm_acc_ peak in the signal, which is due to the impact on the ground. The pre-peak phase is one second long and is related to the falling/descent phase before the impact. The post-peak is also one second long and is related to the dynamics right after the impact. The whole impact phase consists of the pre- and post-peak phases. We then considered a 24.5-s post-impact phase, where resting and (or) recovery can happen. This latter duration was chosen based on the results presented in [11] that disclosed this as the optimal duration to discriminate between falls with, and without, self-recovery.

The overlapping window is one second longer than the CFW (see Figure 1). This additional second is used to perform a peak search. By doing so, we can ensure that there are always enough samples to obtain a CFW, with the desired phases following precise time referencing to the peak. In such a window, the identified reference peak is positioned precisely one second after the start of the window. In cases where two or more peaks are found in the peak search, we select the highest peak.

In the peak search process, we discarded (and did not consider for further analysis) windows with tiny movements. If the peak is below 1.4 g, the windows are directly discarded. This threshold should reflect minimal movements (unlikely to include an impact) that we can directly classify as ADLs without the need for a classifier. The chosen threshold is slightly lower than the one we proposed in previous work (1.5 g [18]) and lower than other thresholds proposed in the literature (e.g., [34] uses thresholds of 1.6 g, 1.8 g, and 1.9 g).

To clarify the whole windowing procedure, we refer to the representative example shown in Figure 1.

The peak search on the first window does not provide any value above the threshold of 1.4 g. So, no CFW is selected. For the second overlapping window, the peak search finds a peak above the threshold instead, and a CFW is selected. The CFW starts one second before the Norm_acc_ peak, includes one second after the peak, and 24.5 additional seconds in the post-impact phase. The third overlapping window presents with an additional peak above 1.4 g. A second CFW is hence selected, with precise time referencing with respect to this new Norm_acc_ peak.

We applied this windowing procedure to the whole dataset of falls and ADLs. The procedure, as shown in the previous example, can identify multiple CFWs in a signal. For falls, this can happen because often there is not a single impact, but rather the fall is characterized by multiple impacts (e.g., first an impact to the chair/wall and then to the ground).

Since we used fall recordings for sensitivity evaluation, we selected a single CFW for a specific fall. To select the most representative one, we retained the CFW nearest to IS, whereas, since we used ADLs for specificity/false alarms evaluations, we retained any possible CFW found in the recording since any CFW could be a false alarm.

We extracted five features from the acceleration signals of the resulting CFW windows.

The segmentation in windows and the feature extraction were both performed automatically. The whole process was performed offline. Each fall and ADL signal were first divided into windows based on the described procedure, and all extracted windows were saved. For every window, feature extraction was performed, and corresponding features were saved. The saved features were then passed to the cross-validation and classification procedure. Table 1 describes the features, and links them to the multiphase fall model. The first three features were also used in a previous study [18], while the last two were added to characterize the post-impact phase.

### 2.5. Classification

To enhance the identification of falls, we combined the information from all features by using several classifiers: Naïve Bayes, logistic regression, KNN, random forests, and SVM. For Naïve Bayes, we used the fitcnb Matlab function. For logistic regression, we used the glmfit and glmval Matlab functions. For KNN, we chose three nearest neighbors and used the fitcknn Matlab function, standardizing the features. For random forests, we selected twenty trees, and we used the TreeBagger function, selecting the regression method to be able to compute the area under the curve (AUC). The SVM classifier was implemented using the LIBSVM library for Matlab [46]. We used the radial basis function kernel and standardized the features.

### 2.6. Training and Testing: Cross-Validation

We performed subject-based cross-validation to evaluate the performance of the classifiers. We opted for five-fold cross-validation.

By performing subject-based cross-validation, all data (both fall and ADL windows) from a single subject was confined to a single specific fold (out of the five) in order to avoid dependencies that could, in turn, decrease the generalizability of the results. In this way, in fact, when training with four of the five folds and testing with the remaining fold, it is assured that data from subjects of the testing fold is only in the testing fold, thus preventing possible overfitting.

The cross-validation was also stratified to account for the variability of the number of falls and ADLs among the subjects. The stratification was performed to have a balanced proportion of subjects with a low/high number of falls and with a low/high number of ADLs within each cross-validation fold.

The subjects with ADLs were divided into low or high, based on the number of their ADLs with respect to the median of the whole group, and were stratified in the five folds based on this division, balancing the proportion of the two groups in each fold and ensuring that all data from a single subject (ADLs and, if available, falls) were in a single fold.

Finally, the remaining subjects (fallers without ADLs) were divided into two groups based on the number of falls (low and high number of falls with respect to the median), and were stratified in the five folds, balancing the proportion of the two groups in each fold and ensuring that all falls of a single subject were in a single fold.

**Figure 2:**
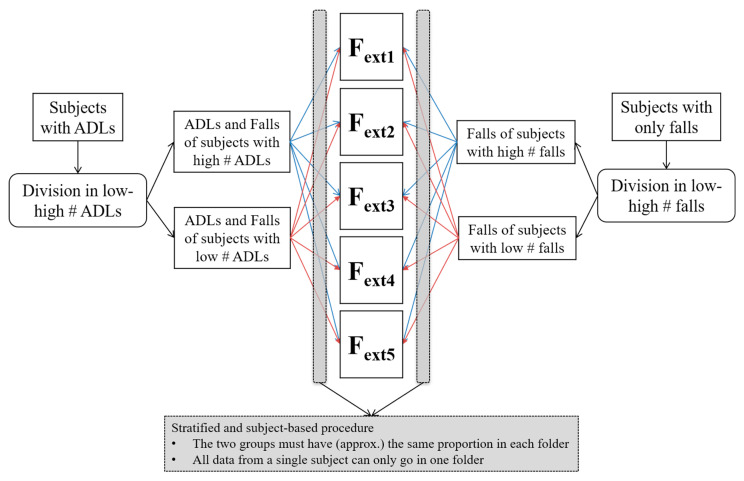
The stratification procedure for the five-fold cross-validation. The suffix “ext” for folds F reflects the fact that this cross-validation for performance evaluation was, in the case of the support vector machine (SVM) classifier, external with respect to internal cross-validation for model parameter selection (see Figure 3).

This procedure (see Figure 2 for a description) was done to avoid dependencies that could limit the generalizability of the results.

**Figure 3:**
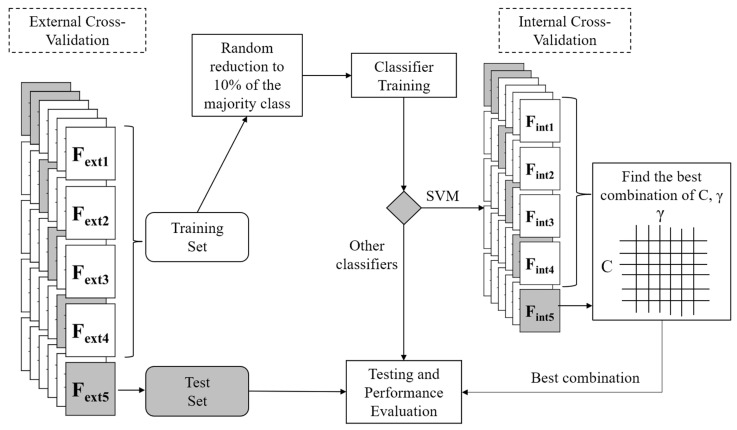
The training and testing procedure for classifiers.

The whole training and testing procedure through cross-validation is shown in Figure 3. Before training the classifiers, in the training set provided by the five-fold cross-validation (composed of four folds), we reduced the samples of the majority class (ADLs) to 10% of its original size by randomly excluding 90% of all ADLs samples in the training set. This was done to compensate for the effect of class imbalance. The dataset, as one expects when analyzing rare (compared to ADLs) events, such as falls, is, in fact, highly unbalanced (see Results section). This reduction was not performed in the testing set. As an additional procedure, only for the SVM classifier, we performed an internal five-fold cross-validation for each run of the above mentioned (external) five-fold cross-validation. So, the internal cross-validation was performed on the training set (composed of four folds) of the external cross-validation. It was performed to search for optimal values of SVM parameters (C and γ). We used a grid-search approach, following the indications in [47]. Pairs of (C, γ) values were evaluated, and the pair with the best cross-validation accuracy was then selected. The search among pairs was done by looking at all possible pairs from exponentially growing sequences of C and γ (C = 2^−5^, 2^−4^, …, 2^15^), and γ = 2^−15^, 2^−14^, …, 2^3^).

### 2.7. Performance Evaluation

The performance of the classifiers was evaluated by computing several measures. Each fall window (in)correctly identified as such was considered as a (false) true positive. Each ADL window (in)correctly identified as such was considered as a (false) true negative.

We computed the receiver operating characteristic (ROC) curves, obtaining area under the curve (AUC) values. Since the AUC does not sufficiently detail the performance of a fall detection system, as suggested by [48], we computed additional performance measures: sensitivity (or recall), specificity, false alarm rate per hour, positive predictive value (or recall), and F-measure. We computed these additional measures, considering a single threshold on the classifier output (whereas AUC considers all possible thresholds). The threshold on the classifier output (which ranges from 0 to 1) was 0.5.

False alarm rate per hour was computed to gain more practical insight into real-world fall detection (i.e., how many false alarms to expect if the system was deployed in practice). This feature was computed by assuming that each false positive (window incorrectly identified as a fall) would result in a false alarm (e.g., emergency contact). It was computed by dividing the total number of false positives for the total number of monitored hours (180 in this study).

To evaluate the added value of the proposed multiphase features, we also compared the classifiers’ performance using the proposed features with the classifiers’ performance using conventional features that are commonly used in fall detection studies [13,25,33,37]. These conventional features were max, min, mean, and standard deviation of *a_x_*, *a_y_*, *a_z_*, and Norm_acc_. Furthermore, we also compared the proposed methodology with the algorithm proposed by Kangas et al. [21], based on multiple thresholds, which is one of the very few studies evaluating performance on real-world falls. It can be exemplified in the following manner. First, a check is made to see if all acceleration signals are below 0.75 g. If this is passed, a check to identify an impact peak on the following samples is made by looking for an acceleration norm over 2 g. If this is also passed, a final check to detect lying posture two seconds after the impact is made. To do this, the vertical acceleration, low-pass filtered at 0.25 Hz, is averaged between 1.6 s after the impact and 2 s after the impact. If this average is less than 0.5 g, a fall is detected.

### 2.8. Software

Matlab R2018b was used for all the analyses in this study. ROC curves and AUC evaluations were implemented using the perfcurve function.

### 2.9. Computational Time Evaluation

The evaluation of the computational time needed by the presented techniques was performed offline using a Dell laptop (Model # M3800, Intel^®^ Core™ i7-4712HQ, CPU @2.30 Gz, 16 GB RAM, 64-bit Windows 10 Pro).

## 3. Results

We passed to the classifier stage a total of 143 fall windows and 20,783 ADL windows.

None of the recorded falls exhibited peaks in Norm_acc_ lower than the 1.4 g threshold, with the lowest peak observed at 1.6 g, confirming that we had selected an appropriate threshold.

For both falls and ADLs, the number of instances significantly differed across subjects. There was a median of 2 falls per subject, ranging from 1 to 23. There was a median of 551 ADLs per subject, ranging from 3 to 8503.

According to the FRAT-up tool, the fall risk of subjects analyzed for falls and ADLs was similar: 0.44 ± 0.06 and 0.42 ± 0.04, respectively.

Table 2 shows the performance of the classifiers used in this study with multiphase or conventional features. All classifiers performed better with the features based on the multiphase fall model. This is well documented by the F-measure values, which are always higher when the multiphase features are used. Regarding AUCs, these are also higher with multiphase features, with the only exception of KNN, where the AUC value is slightly higher (but very similar) for the conventional features.

The AUC value for the algorithm by Kangas et al. is not presented, since the algorithm only produces a binary output.

## 4. Discussion

In this study, we analyzed real-world data (falls and ADLs) to implement and evaluate machine learning algorithms for fall detection based on acceleration features extracted from a multiphase fall model.

A new overlapping windowing technique was designed, which provides windows for feature extraction with precise time referencing to the peak of the Norm_acc_ signal. This technique was developed to identify falls from acceleration signals, but could be applied to other biomedical signals when one desires windows with precise time referencing to a pivotal point of the signal. Putra et al. [34] recently proposed an event-triggered machine learning approach in fall detection that has similarities with the proposed approach. Still, in our approach, we do not propose a technique that is an alternative to overlapping windows (as Putra et al. do), but instead we propose a technique that uses standard overlapping windows to extract features with precise time referencing. Also, we extract features characterizing the specific phase, while Putra et al. extract the same standard features for each phase.

A noteworthy property of our proposed feature set is that it does not depend on the orientation of the sensor, since all features are extracted from the acceleration norm. This choice ensures a solution with higher reliability, wearability, and usability.

There was high variability in the number of selected ADL windows among different subjects. This variability reflected the varying levels of physical activity (i.e., if a subject stood or sat all day, the acceleration norm would rarely exceed the 1.4 g threshold). In fallers, the number of falls per subject was also very heterogeneous, because subjects were recorded for different periods and had different intrinsic fall risks. We addressed this issue when designing the cross-validation procedure.

When considering the obtained performance results, features from the multiphase model overperformed conventional features for each classifier, both in F-measure and AUC values. The only exception was related to AUC values from KNN, which were very similar between the two feature sets (slightly higher for conventional features). Regarding this, it should also be noted that KNN AUC values were lower than AUCs obtained by other classifiers.

Features from the multiphase model, with any classifier, overperformed the F-measure of the threshold-based method. Regarding AUC performance in general, we can see that all classifiers using multiphase features had very similar values, very close to the “optimal” one.

Still, an AUC that seems optimal (0.996 as an example) translates into combinations of sensitivity and false alarm rates that are still far from optimal (88% of sensitivity with more than one false alarm per hour is not usable in any real-world context, because of too many false alarms). It is clear from these results that the AUC metric does not suffice in order to detail the performance of a fall detection system, confirming what was suggested in a recent scoping review [48]. The AUC is indeed a global metric that evaluates the overall performance of the classifier for every possible threshold on the values of the output scores (each threshold corresponding to a combination of sensitivity and specificity). However, in the context of the real-world functioning of a fall detection system with automatic alarm and response capabilities, AUC alone fails to provide enough information on its performance. Moreover, considering a fall detection system in its daily operation, a single trade-off must, in the end, be chosen between sensitivity and specificity (false alarms). Although one may desire a system that is very sensitive, i.e., is accurate in detecting falls, the associated number of false alarms may become so bothersome that the user of the system would quit altogether.

Based on a single threshold, we reported sensitivity, specificity, false alarm rate per hour, positive predictive value, and F-measure. Regarding the latter, it is a measure that takes into account the trade-off between sensitivity (or recall) and positive predictive value (or precision), being the harmonic mean of the two. It was proposed as the single standard measure to use for comparison of fall detection systems by [48]. In our sample, the best F-measure was obtained by support vector machines learning from the multiphase features, with a 64.6% value, overperforming the second-best method (logistic regression with multiphase features), which has a 59.8% value.

Again in [48], it was suggested to report recall and precision, together with the F-measure, to obtain a detailed global view of the fall detection performance. These measures are indeed useful. However, we also reported the false alarm rate (per hour in the present study), since we believe this to be an evident, practical, and useful measure to evaluate a fall detection system when the aim is to deploy it in the real-world. This is because false alarms are a convenient and understandable measure of usability. This is clear from Table 2, where a specificity that could be considered nearly optimal in several fields (95.5%) corresponds to a false alarm rate (more than five false alarms per hour) that would not be acceptable for practical use in fall detection in any scenarios. False alarm rate, in conjunction with sensitivity, can indeed provide a clear picture of the two practical aspects that matter most in acceptability of the real-world fall detection system: how many falls will be correctly detected, and how many false alarms the user will have to cope with. The sensitivity we obtained for the most promising method in our study, SVM with multiphase features, seems already acceptable for real-world use (over 80%), but the false alarm rate would still not be. More work will be needed on this huge open issue, since one false alarm every two monitored hours is indeed too much. It is then apparent in our opinion that all studies proposing a method for fall detection should always report false alarm rates in their performance evaluation, together with the other three measures suggested above: sensitivity (recall), positive predictive value (precision), and F-measure.

### 4.1. Limitations

Although falls were identified via visual inspection by two experienced raters [28], fall reports did not always align perfectly with the patterns observed in the signals. So, it is conceivable that some falls were incorrectly identified. In addition to misreporting, unreported falls (underreporting by the subjects) may also be among the ADLs. Both issues (mis- and underreporting) might influence and negatively impact the classifier’s ability to learn patterns. For instance, in the case of underreporting, the algorithm would learn a pattern of an ADL that is a fall instead.

### 4.2. Implications for Fall Detection Systems and Clinical Research

It is clear that even the most favorable of the proposed methods has a false alarm rate that would not be acceptable for a marketable fall detection product. Still, our objective in this paper was to analyze the performance of state-of-the-art machine learning algorithms on conventional features and newly proposed features on a relatively large database of real-world falls. Features based on a multiphase fall model consistently overperformed conventional features. Choosing additional features based on this model or other classifiers (e.g., deep learning), increasing the dataset sample size, and analyzing different signals (e.g., angular velocities) are all options that could further improve the performance obtained in this study.

The full deployment of a real-world effective fall detection algorithm could directly impact clinical research, besides impacting the quality of related emergency assistance. In fact, most observational and intervention studies rely on self-report to acquire prospective falls information (e.g., the number of falls endured by a given participant). The reliability of this information remains questionable, due to the biases innate in reporting [49,50] and recall [51,52], especially for older individuals in which aspects of compromised memory often come into play. The use of objective measures (e.g., the number of falls detected automatically) would enable a more accurate and reliable evaluation of the number of falls endured and, consequently, would improve the overall quality of fall-related studies.

As another clinical insight, some of the falls had an immediate recovery (a few seconds) and, therefore, a very short resting phase post-impact [11]. A future improvement could stem from differentiating falls with immediate recovery, falls with recovery after a certain period, and falls with no recovery at all (i.e., long lie). Automatically identifying the type of recovery could help to choose the proper response [11]. For example, not identifying a fall with immediate recovery would be less dangerous than not identifying a fall resulting in a long lie. An approach based on the automatic identification of different severity levels after the fall could help in this perspective, as proposed in [53]. This concept should also be implemented in the learning and evaluation of fall detection algorithms. For this to be possible, every fall would need a detailed and reliable report on the presence/absence of recovery (which was not the case for some of the falls present in this study). A further improvement could be made by differentiating the consequences of the falls (e.g., injuries or need for medical care).

The choice of the proper trade-off between sensitivity and false alarms can also depend on the purpose of the device (or service model). A maximum sensitivity would be chosen for a research application, since positive signals are typically verified or falsified by phone calls from research staff. On the other hand, an emphasis on false alarms would be applied to home alarm systems when users are not willing to accept a high rate of false alarms. In Table 3, to gain practical insight into this trade-off, five different representative thresholds are chosen for the classifier’s output of the most promising method. At one end, the classifier can arrive at almost 100% sensitivity, at the expense of increasing false alarms to very high values (more than five per monitored hour). At the other end, having approximately one false alarm every 20 monitored hours is associated with correctly detecting only one out of two falls.

False alarm rates should be reported to increase comparability across different studies, and to provide a more practical measure of performance.

The current study analyzed an unprecedented number of real-world falls, compared to previous studies. Still, more falls are needed for machine learning algorithms to learn fall patterns effectively. One potential option to increase the number of fall signals collected in real-world settings would be crowdsourcing via smartphone apps, which could exponentially increase the access to fall signals.

Regarding the hardware, in this study, smartphones or dedicated wearable systems were used. As a different research area, the incorporation of fall detection systems into existing electronic devices (such as hearing aids, cochlear implants, or pacemakers) could be an unobtrusive way to detect falls. It is essential to evaluate the computational cost when considering the actual feasibility and implementation of a fall detection algorithm using a wearable unit of this type [54]. In general, this is an aspect that is very important when deploying a system in the real world, considering the available computational capabilities of the chosen device (e.g., smartphone or dedicated sensors). We report in Table 4 a preliminary analysis in this direction. It should be noted that the evaluations were performed offline. The computational time needed for data acquisition (from the raw signal to the window ready for feature extraction), feature extraction, and classification were computed and are reported in Table 4. As expected, data acquisition takes longer (but still well below one millisecond) for the multiphase features with respect to the other options. Multiphase feature extraction is also higher with respect to conventional features, but lower than features from Kangas et al. (probably because of the filtering that is present in this method to detect posture). Classification with random forests has a computational time much higher than any other classifier. On the other hand, as expected, the threshold-based method had the fastest classification. The method that showed the most promising performance in fall detection (SVM with multiphase features) has a total computational time which is less than one millisecond (0.7 ms).

As a further note, we obtained our results investigating falls and ADLs from populations with moderate-to-high fall risk. Whether (and how) these results would transfer to a healthy older people population (or to other populations) should need further testing. Regarding the validation of fall detection algorithms, it is interesting to note that the threshold-based method shows a sharp decrease in performance with respect to the results reported in the original study (80% sensitivity, 0.049 false alarm rate per hour), where it was also tested on real-world falls. This highlights the importance of external validation of fall detection algorithms, and the importance of reporting comparable performance measures across studies.

## 5. Conclusions

In this study, we exploited the unique real-world fall data from the FARSEEING fall repository to train and test machine learning algorithms for fall detection based on acceleration signals recorded by a single wearable sensor. The implemented algorithms learning from features based on a multiphase model overperformed algorithms learning on conventional features. Finally, we provided and suggested useful metrics for characterizing fall detection systems to deploy in the real world.

## Figures and Tables

**Table 1:** Extracted features.

Feature	Description	Phase
Lower peak value (LPV)	Minimum value of Norm_acc_ before the identified peak.	Pre-peak
Upper peak value (UPV)	Value of the identified peak of Norm_acc_. It is, by construction, the value of the signal 1 s after the start of the window.	Peak sample
Wavelet-based coefficient	A mother wavelet was defined as the average of the fall signals in the training set. A similarity coefficient to such mother wavelet was calculated following the procedure described in [18].	Impact (pre-peak + post-peak)
Periodicity after impact	This feature is based on the autocorrelation of Norm_acc_. The rationale is that in the interval right after a fall, there cannot be a periodic movement, such as walking or running. It is computed in a segment of 2 s, starting 0.5 s after the peak sample.	Post-peak and post-impact
Standard deviation after impact	Standard deviation (SD) of Norm_acc_ during the post-impact phase. The rationale is that in the interval following the impact, the subject might produce limited movements compared to normal ADLs.	Post-impact

**Table 2:** Classification results.

Classifier	Features	AUC	Sensitivity (Recall) [%]	Specificity [%]	False Alarm Rate [FA/hour]	Positive Predictive Value (Precision) [%]	F-Measure [%]
Naïve Bayes	MultiPhase	0.996	88.1	99.1	1.09	39	54.1
Logistic Regression	MultiPhase	0.996	83.2	99.3	0.76	46.6	59.8
KNN	MultiPhase	0.958	83.9	99.2	0.92	42.1	56.1
Support Vector Machines	MultiPhase	0.993	81.1	99.5	0.56	53.7	64.6
Random Forests	MultiPhase	0.989	83.2	98.9	1.32	33.3	47.6
Naïve Bayes	Conventional	0.977	95.1	95.5	5.23	12.6	22.3
Logistic Regression	Conventional	0.987	84.6	98.5	1.7	28.3	42.5
KNN	Conventional	0.959	85.3	98.8	1.42	32.4	46.9
Support Vector Machines	Conventional	0.986	83.9	98.6	1.61	29.3	43.4
Random Forests	Conventional	0.985	88.8	98.6	1.57	31	45.9
Threshold-based	Kangas et al.	/	30.1	99.3	0.82	22.9	26.3

**Table 3:** Additional combinations of sensitivity and false alarm rates.

SVM with Multiphase Features					
Sensitivity [%]	53.8	64.3	81.1	93.7	95.1
FA rate [FA/h]	0.06	0.11	0.56	2.78	5.56

Five thresholds are chosen so that false alarm (FA) rates are, from the left: one-tenth and one-fifth of the one reported in Table 2, the same as the reported one, and five and ten times the reported one.

**Table 4:** Computational time for a single window.

Classifier	Features	Data Acquisition (ms)	Feature Extraction (ms)	Classification (ms)
Naïve Bayes	MultiPhase	0.145 (0.07)	0.104 (0.05)	0.695 (0.15)
Logistic Regression	MultiPhase	0.145 (0.07)	0.104 (0.05)	0.03 (0.01)
KNN	MultiPhase	0.145 (0.07)	0.104 (0.05)	1.265 (0.23)
Support Vector Machines	MultiPhase	0.145 (0.07)	0.104 (0.05)	0.452 (0.14)
Random Forests	MultiPhase	0.145 (0.07)	0.104 (0.05)	17.63 (5.1)
Naïve Bayes	Conventional	0.007 (0.01)	0.049 (0.05)	0.686 (0.44)
Logistic Regression	Conventional	0.007 (0.01)	0.049 (0.05)	0.065 (0.05)
KNN	Conventional	0.007 (0.01)	0.049 (0.05)	1.176 (0.28)
Support Vector Machines	Conventional	0.007 (0.01)	0.049 (0.05)	0.368 (0.26)
Random Forests	Conventional	0.007 (0.01)	0.049 (0.05)	16.166 (31.06)
Threshold-based	Kangas et al.	0.008 (0.03)	0.54 (0.15)	0.002 (0.02)
